# Molecular Mechanism of the IRF1/NFE2L1‐DT/ALKBH5/Cx43 Axis in Radiation‐Induced Injury in Vascular Endothelial Cells Through Pyroptosis

**DOI:** 10.1002/kjm2.70159

**Published:** 2025-12-24

**Authors:** Chen Li, Jia‐Wen Yang, Yong‐Rui Jia, Jie Zhang, Qiao Gou

**Affiliations:** ^1^ Key Laboratory of Radiological Protection and Nuclear Emergency, China CDC National Institute for Radiological Protection, Chinese Center for Disease Control and Prevention Beijing China; ^2^ Medical and Health Analysis Center Peking University Beijing China; ^3^ University of Science and Technology of China Hefei China; ^4^ High Magnetic Field Laboratory, Key Laboratory of High Magnetic Field and Ion Beam Physical Biology, Chinese Academy of Sciences; Anhui Province Key Laboratory of Environmental Toxicology and Pollution Control Technology Hefei Institutes of Physical Science, Chinese Academy of Sciences Hefei China

**Keywords:** ALKBH5, Cx43, IRF1, NFE2L1‐DT, PELP1

## Abstract

Radiotherapy effectively eradicates tumor cells but can also trigger pyroptotic damage in vascular endothelial cells. This study investigates the role of interferon regulatory factor 1 (IRF1) in radiation‐induced endothelial injury, aiming to provide mechanistic insights for optimizing radiotherapy. Human umbilical vein endothelial cells (HUVECs) were exposed to x‐ray irradiation, after which cell viability, lactate dehydrogenase (LDH) release, γ‐H2AX foci formation, and the expression of pyroptosis‐associated proteins (NLRP3, Cleaved Caspase‐1, GSDMD‐N, IL‐1β, IL‐18) were assessed. Expression levels of IRF1, NFE2L1‐DT, PELP1, ALKBH5, and Cx43 were quantified. Chromatin enrichment of IRF1 at the NFE2L1‐DT promoter and IRF1–NFE2L1‐DT interactions were examined, along with NFE2L1‐DT or ALKBH5 binding to PELP1. The enrichment of m^6^A modifications on Cx43 transcripts was also evaluated. X‐ray exposure reduced HUVEC viability, elevated LDH release, increased γ‐H2AX foci, and upregulated IRF1, along with pyroptosis markers. Silencing IRF1 reversed these changes. Mechanistically, IRF1 directly bound to and increased NFE2L1‐DT expression. NFE2L1‐DT interacted with PELP1 to enhance the binding of PELP1 to and ALKBH5 mRNA, thus upregulating ALKBH5 expression. ALKBH5‐mediated m^6^A demethylation subsequently downregulated Cx43 expression. Overexpression of NFE2L1‐DT or ALKBH5, or silencing Cx43, attenuated the protective effects of IRF1 silencing against radiation‐induced damage. These findings indicate that radiation‐induced IRF1 upregulation leads to endothelial injury by promoting pyroptosis through the NFE2L1‐DT/ALKBH5/Cx43 axis.

## 1 Introduction

Radiotherapy remains a cornerstone of cancer therapy, employing ionizing radiation (IR) to induce DNA damage, arrest cell proliferation, and eradicate malignant cells [1]. However, inadvertent exposure of normal tissues, particularly vascular endothelial cells, to IR can provoke cellular senescence and apoptosis, contributing to radiation‐associated cardiovascular complications [2]. Further, IR induces endothelial hyperpermeability, basement membrane disruption, and pro‐inflammatory signaling, which together amplify oxidative stress and drive fibrotic remodeling [3]. Although advances in radiotherapy techniques and adjunctive pharmacological strategies have reduced off‐target toxicity [4], residual radiation‐induced injury remains a significant clinical challenge. Understanding the molecular mechanisms underlying radiation‐mediated endothelial damage is, therefore, a critical area of investigation.

Interferon regulatory factor 1 (IRF1), an evolutionarily conserved transcription factor of the IRF family, plays a pivotal role in innate immunity and cellular homeostasis [5]. Increasing evidence implicates IRF1 activation in endothelial senescence, apoptosis, and impaired regenerative capacity, ultimately contributing to organ dysfunction [6, 7]. IR exposure has been shown to upregulate IRF1 transcription, initiating pro‐inflammatory signaling and immune dysregulation [8]. IRF1 silencing attenuates ferroptosis in intestinal epithelial cells and mitigates radiation‐induced intestinal injury [9]. However, the precise mechanisms through which IRF1 contributes to endothelial cell injury following radiation remain incompletely understood.

Long non‐coding RNAs (lncRNAs), transcribed by RNA polymerase II, are increasingly recognized as regulators of genome stability. Dysregulated lncRNA expression can induce transcription–replication conflicts and R‐loop‐associated DNA damage [10]. Aberrant lncRNA profiles have been reported in radiation‐induced vascular endothelial injury, as exemplified by PVT1 [11] and lncRNA E230013L22Rik [12]. NFE2L1‐DT, a recently identified lncRNA, shows transcriptional upregulation in endothelial cells following x‐ray exposure [13]. Transcription factors can increase lncRNA stability via promoter binding [14]. Based on this evidence, we hypothesize that IRF1 may regulate radiation‐induced endothelial injury through direct binding to and stabilization of NFE2L1‐DT.

N6‐methyladenosine (m^6^A) is the most prevalent and evolutionarily conserved internal modification in eukaryotic RNA, regulating gene expression by reversible deposition through m^6^A methyltransferases and removal by demethylases [15]. AlkB homolog 5 (ALKBH5), a major endogenous m^6^A demethylase, influences pre‐mRNA splicing, mRNA stability, and translational efficiency within RNA metabolic pathways [16]. ALKBH5 deficiency has been reported to attenuate DNA damage induced by x‐ray irradiation [17]. Proline‐, glutamic acid‐, and leucine‐rich protein 1 (PELP1), a multifunctional scaffold protein, serves as a regulatory hub for diverse transcription factors and nuclear receptors [18]. Computational prediction suggests a potential binding interaction between NFE2L1‐DT and PELP1, and PELP1 has been shown to interact with ALKBH5. These findings prompted us to explore the contribution of the NFE2L1‐DT/ALKBH5 axis to radiation‐induced endothelial injury.

Connexin 43 (Cx43) is synthesized in the endoplasmic reticulum, oligomerized in the Golgi apparatus, and transported to the plasma membrane as closed hemichannels, where it mediates intercellular communication via gap junctions [19]. In human umbilical vein endothelial cells (HUVECs), Cx43 expression is significantly reduced during x‐ray‐induced pyroptosis [20]. Based on these observations, we hypothesized that IRF1 may contribute to radiation‐induced injury through regulation of the NFE2L1‐DT/ALKBH5/Cx43 signaling axis.

In this study, we established an HUVEC radiation injury model using x‐ray exposure to delineate the molecular mechanisms by which the IRF1/NFE2L1‐DT/ALKBH5/Cx43 pathway regulates pyroptosis. Our findings aim to provide novel mechanistic insights and potential therapeutic strategies for mitigating adverse vascular effects during cancer radiotherapy.

## 2 Materials and Methods

### 2.1 Cell Culture and Transfection

HUVECs (PCS‐100‐010, ATCC, Manassas, VA, USA) were cultured in Endothelial Cell Growth Kit medium (PCS‐100‐040, ATCC) following the manufacturer's instructions and maintained at 37°C in a humidified atmosphere containing 5% CO_2_. Upon reaching approximately 70% confluence, cells were exposed to x‐ray irradiation at doses of 0, 4, 8, 10, 14, or 18 Gy at room temperature using an RS2000 irradiator (Rad Source, Suwanee, GA, USA). Cells were harvested at 0, 6, 24, and 48 h postirradiation. The dose–time combination that most consistently produced reproducible injury was selected to establish the irradiation (IR) group.

Full‐length sequences of NFE2L1‐DT and ALKBH5 were cloned into the pcDNA3.1‐EGFP expression vector (HG‐VPH0002, HonorGene, Hunan, China) to generate overexpression constructs (oe‐NFE2L1‐DT, oe‐ALKBH5). HUVECs were seeded into 96‐well plates and transfected at approximately 80% confluence with siRNA or plasmid constructs, including si‐NC, si‐IRF1, oe‐NC, oe‐NFE2L1‐DT, oe‐ALKBH5, and si‐Cx43, using Lipofectamine 3000 (L3000150, Thermo Fisher Scientific, Waltham, MA, USA). Each transfection group was prepared in multiple replicates. After 48 h of incubation, cells were collected for subsequent analyses. All siRNAs and expression constructs were synthesized by GenScript (Nanjing, Jiangsu, China).

### 2.2 Cell Viability Assay

HUVECs were seeded in 96‐well plates at a density of 8 × 10^3^ cells per well and incubated for 24 h. Following this, 10 μL of CCK‐8 reagent (K1018, ApeBio, Houston, TX, USA) was added to each well, followed by incubation at 37°C for 2 h. Absorbance was then recorded at 450 nm using a Varioskan ALF microplate reader (VA000010C, Thermo Fisher Scientific).

### 2.3 Lactate Dehydrogenase (LDH) Release Assay

Cells were allocated into three groups: blank control (untreated cells), test sample (treated cells), and maximum enzyme activity control (untreated cells exposed to lysis buffer). Cells were seeded in 96‐well plates at a density of 1 × 10^4^ cells per well and incubated overnight. LDH release was quantified using a commercial kit (C20300, Thermo Fisher Scientific) following the manufacturer's protocol. LDH release was calculated as:

$$\mathrm{LDH}\;\text{release}\;\left(\%\right)=\frac{{\mathrm{OD}}_{\text{test}}-{\mathrm{OD}}_{\text{blank}}}{{\mathrm{OD}}_{\max}-{\mathrm{OD}}_{\text{blank}}}\times 100$$

### 2.4 Immunofluorescence

HUVECs were fixed with 4% paraformaldehyde (P0099, Beyotime, Shanghai, China) and permeabilized with 0.1% Triton X‐100 (P0096, Beyotime), followed by washing with PBS. After blocking with bovine serum albumin (ST023, Beyotime) for 30 min, cells were incubated overnight at 4°C with a γ‐H2AX primary antibody (ab81299, 1:250, Abcam, Cambridge, UK). The cells were then incubated with an IgG secondary antibody (ab150079, 1:1000, Abcam) for 1 h at 37°C. Nuclear staining was performed using 4′,6‐diamidino‐2‐phenylindole (DAPI; C1002, Beyotime), and fluorescence imaging was captured using an FV3000 confocal laser scanning microscope (Olympus, Tokyo, Japan). Quantitative analysis of γ‐H2AX foci was performed with ImageJ software (NIH, Bethesda, MD, USA).

### 2.5 Western Blot

Successfully transfected HUVECs were harvested, and total protein was extracted using radioimmunoprecipitation assay (RIPA) buffer (89901, Thermo Fisher Scientific). Proteins were separated by sodium dodecyl sulfate–polyacrylamide gel electrophoresis (SDS‐PAGE) and transferred onto polyvinylidene fluoride (PVDF) membranes (88520, Thermo Fisher Scientific). After blocking with 5% skim milk, membranes were incubated overnight at 4°C with primary antibodies against NLRP3 (ab263899, 1:1000, Abcam), Cleaved Caspase‐1 (89332, 1:1000, Cell Signaling, Danvers, MA, USA), GSDMD‐N (ab215203, 1:1000, Abcam), IRF1 (ab243895, 1:1000, Abcam), PELP1 (ab200203, 1:1000, Abcam), ALKBH5 (ab195377, 1:1000, Abcam), Cx43 (ab217676, 1:1000, Abcam), and GAPDH (ab181602, 1:10000, Abcam). After washing, membranes were incubated for 1 h at room temperature with secondary antibodies (goat anti‐rabbit IgG, ab205718, 1:10000, Abcam; goat anti‐mouse IgG, ab205719, 1:2000, Abcam). Protein bands were visualized using enhanced chemiluminescence (ECL) reagent (34580, Thermo Fisher Scientific) and quantified using ImageJ software (NIH, Bethesda, MD, USA).

### 2.6 Enzyme‐Linked Immunosorbent Assay (ELISA)

Successfully transfected HUVECs were harvested and seeded into 6‐well plates at a density of 5 × 10^4^ cells/well. Following incubation, cell culture supernatants were collected and clarified by centrifugation at 4°C. The concentrations of interleukin (IL)‐18 and IL‐1β were measured using ELISA kits (D711091/D711068, Sangon, Shanghai, China) following the manufacturer's instructions.

### 2.7 Reverse Transcription Quantitative Polymerase Chain Reaction (RT‐qPCR)

Total RNA was isolated using RNAiso reagent (9109, Takara, Tokyo, Japan) and reverse‐transcribed into cDNA with the HiScript II 1st Strand cDNA Synthesis Kit (R212, Vazyme, Nanjing, Jiangsu, China). Quantitative real‐time PCR (RT‐qPCR) was conducted using SYBR GreenER qPCR SuperMix (11762500, Thermo Fisher Scientific) on a Stratagene MX4000 system (Reuzeit, Temecula, CA, USA), with GAPDH serving as the internal control. Relative transcript levels were determined by the 2^−ΔΔCt^ method [21]. Primers are listed in Table 1.

**TABLE 1:** PCR primer sequences.

Gene	Sequences (5′–3′)
IRF1	F: GAAGGCCAACTTTCGCTGTG
	R: CCAGGTGGCATCCATGTTCT
NFE2L1‐DT	F: ATAGGAAGGTGTATCGCCGC
	R: ACGCGTCAGTAGTCCCTGAT
PELP1	F: CTCTGAGTTTGGAGCTCCCG
	R: CTTCCCCCACATCCAGCTTT
ALKBH5	F: CTCTTCAGCCAGGACGAGTG
	R: CCAACCGGGGTGCATCTAAT
Cx43	F: GGCCTTCTTGCTGATCCAGT
	R: GCTGGTCCACAATGGCTAGT
GAPDH	F: TGCACCACCAACTGCTTAGC
	R: GGCATGGACTGTGGTCATGAG

### 2.8 Bioinformatics

The JASPAR database (http://jaspar.genereg.net/) [22] was used to predict the binding sites of IRF1 in the lncRNA NFE2L1‐DT promoter region. The RNAct database (http://rnact.crg.eu) [22] was employed to predict the binding relationship between NFE2L1‐DT and PELP1 protein. The RPISeq database (http://pridb.gdcb.iastate.edu/RPISeq/) [23] was used to predict the binding probability between the PELP1 protein and ALKBH5. Predictions from the RPISeq database are generated using Random Forest (RF) and Support Vector Machine (SVM) classifiers. A predicted interaction is considered positive when both RF and SVM scores exceed 0.5, indicating a potential binding relationship between the two molecules.

### 2.9 Chromatin Immunoprecipitation (ChIP) Assay

ChIP was conducted using the SimpleChIP Plus Sonication ChIP Kit (56383, Cell Signaling). HUVECs were crosslinked with 1% formaldehyde, and the reaction was quenched with glycine. Following cell lysis, chromatin was fragmented by sonication and incubated overnight at 4°C with either IRF1 primary antibody (ab243895, Abcam) or control IgG (ab6757, Abcam). Protein A/G magnetic beads were added to capture immune complexes. After sequential washes with ChIP buffer, DNA–protein complexes were eluted with sodium bicarbonate solution and reverse‐crosslinked. The purified DNA was recovered using the FastPure Gel DNA Extraction Mini Kit (DC301, Vazyme) and quantified by RT‐qPCR.

### 2.10 Dual‐Luciferase Reporter Assay

Wild‐type (WT) and mutant (MUT) NFE2L1‐DT promoter sequences containing the IRF1 binding site were cloned into the pGL4.21 plasmid (EC761, Promega, Madison, WI, USA) to generate promoter‐WT and promoter‐MUT constructs. Cells were co‐transfected with either the IRF1 overexpression vector or the empty control vector along with promoter‐WT or promoter‐MUT plasmids. After 48 h, cells were lysed, and luciferase activity was measured using the Dual‐Glo Luciferase Assay System (E2940, Promega).

### 2.11 RNA Immunoprecipitation (RIP) Assay

Cells were lysed in RIP lysis buffer supplemented with protease and RNase inhibitors. Protein A/G magnetic beads were preincubated with either anti‐PELP1 (96986, Cell Signaling) or anti‐IgG (ab172730, Abcam) antibodies for 4 h at room temperature, following the manufacturer's protocol for the RNA Immunoprecipitation Kit (17‐700, Sigma‐Aldrich, Saint Louis, MO, USA). The antibody–bead complexes were then incubated with cell lysates overnight at 4°C. After washing, the bound RNA was purified and analyzed by RT‐qPCR.

### 2.12 RNA Pull‐Down Assay

The RNA pull‐down assay was performed using the Smart‐RNA‐Pull RNA Pull‐Down Kit (IF9608, Engibody, Milwaukee, WI, USA). A portion of the cell lysate was reserved for Western blot analysis as the input control, while the remaining lysate was incubated with either a biotin‐labeled NFE2L1‐DT probe (Bio‐NFE2L1‐DT) or a negative control probe (Bio‐NC). RNA–protein complexes were captured using streptavidin magnetic beads, and PELP1 expression in each group was subsequently assessed by Western blot.

### 2.13 RNA Stability Assay

HUVECs were seeded into 6‐well plates and incubated overnight, followed by treatment with actinomycin D (HY‐17559, MCE, Monmouth Junction, NJ, USA) for 0, 2, 4, or 8 h. The mRNA stability of ALKBH5 and Cx43 was subsequently assessed by RT‐qPCR.

### 2.14 Total m^6^A Quantification

Total m^6^A levels were quantified using the EpiQuik m^6^A RNA Methylation Quantification Kit (P‐9005, Epigentek, Farmingdale, NY, USA). Cellular RNA was extracted with RNAiso reagent (9109, Takara), and its concentration was measured. Working solutions were prepared following the manufacturer's instructions, and absorbance at 450 nm was recorded. The m^6^A content in total RNA was calculated based on a standard curve.

### 2.15 Methylated RNA Immunoprecipitation‐qPCR (MeRIP‐qPCR) Analysis

The m^6^A methylation status of Cx43 was assessed using the Magna MeRIP m^6^A Kit (17‐10499‐2, Sigma‐Aldrich). Cellular RNA was fragmented in RNA fragmentation buffer and isolated. Magnetic beads were conjugated with either anti‐m^6^A antibody (ab208577, Abcam) or IgG control (5415, Cell Signaling) and incubated with the fragmented RNA at 4°C for 2 h. Following washing and RNA purification, m^6^A enrichment was quantified by RT‐qPCR.

### 2.16 Statistical Analysis

Data analysis and visualization were performed using SPSS 21.0 (IBM SPSS Statistics, Chicago, IL, USA) and GraphPad Prism 8.0 (GraphPad Software Inc.). Continuous variables are expressed as mean ± standard deviation. Normality and homogeneity of variance were assessed before statistical testing. For data meeting the assumptions of normal distribution and equal variance, comparisons between two groups were conducted using *t*‐tests, while multiple group comparisons were analyzed using one‐way or two‐way analysis of variance (ANOVA) followed by Tukey's post hoc test. All *p*‐values were two‐tailed, with *p* < 0.05 considered statistically significant.

## 3 Results

### 3.1 Silencing IRF1 Alleviates Radiation‐Induced Injury in HUVECs by Inhibiting Pyroptosis

**FIGURE 1:**
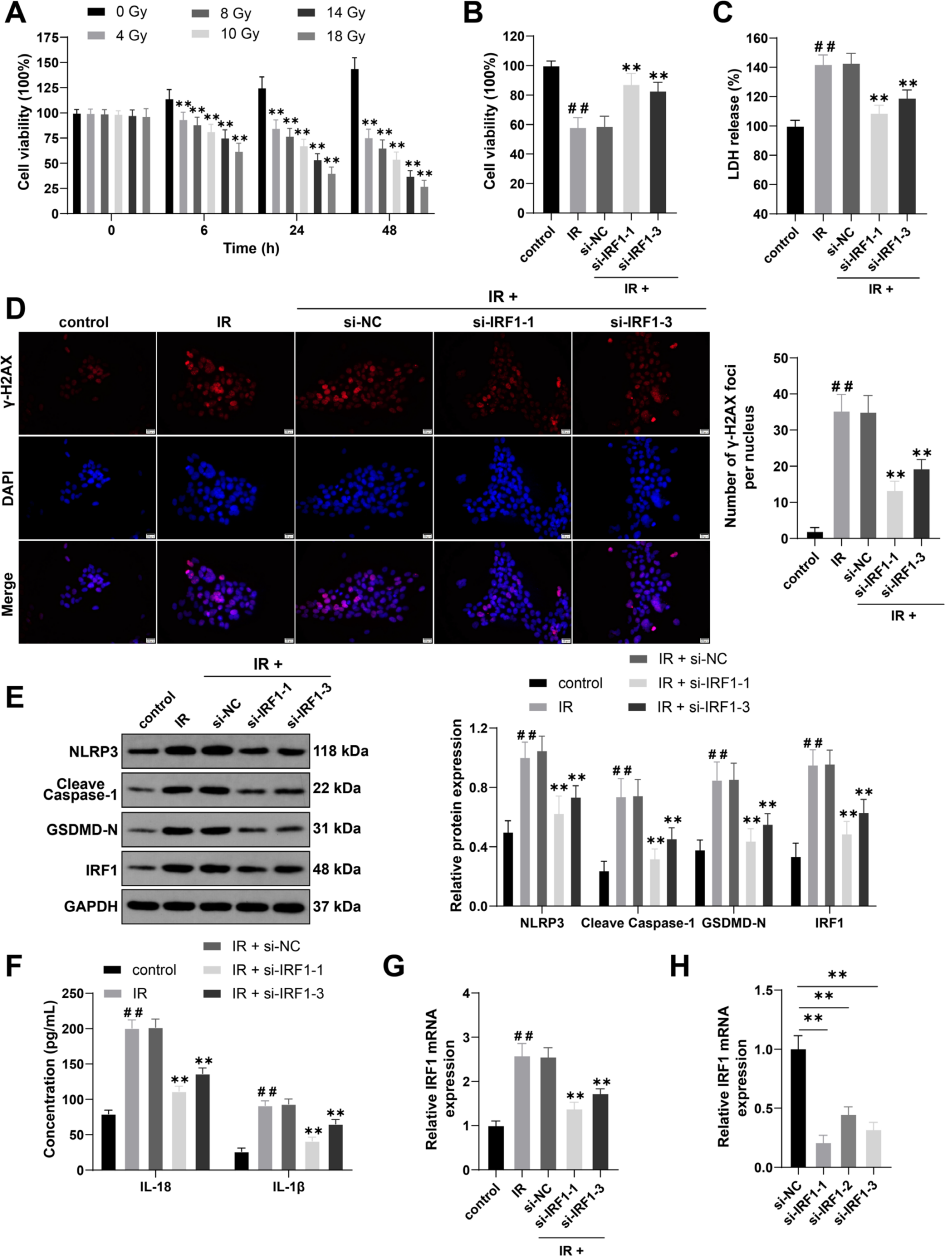
Silencing IRF1 mitigates radiation‐induced injury in HUVECs by suppressing pyroptosis. HUVECs were exposed to graded doses of x‐rays (0, 4, 8, 10, 14, 18 Gy) for different durations (0, 6, 24, 48 h). (A) Cell viability was assessed by CCK‐8 assay. HUVECs treated with 10 Gy x‐rays for 48 h were selected as the IR group for subsequent experiments. Three siRNAs targeting IRF1 were transfected into HUVECs, with si‐NC as a control. (B) Cell viability was evaluated by the CCK‐8 assay. (C) LDH release was quantified using a commercial kit. (D) DNA double‐strand breaks were detected by γ‐H2AX immunofluorescence. (E) Protein expression of NLRP3, Cleaved Caspase‐1, GSDMD‐N, and IRF1 was analyzed by Western blot. (F) IL‐1β and IL‐18 levels were measured by ELISA. (G and H) IRF1 expression was quantified by RT‐qPCR. Data represent mean ± standard deviation from three independent experiments. For panels A, E, and F, multiple group comparisons were analyzed by two‐way ANOVA; for panels B–D, G, and H, one‐way ANOVA followed by Tukey's post hoc test was applied. Statistical significance: (A) **p < 0.01 versus 0 Gy; (B–G) ##*p* < 0.01 versus control group; **p < 0.01 versus IR + si‐NC group; (H) **p < 0.01 versus si‐NC.

The HUVECs were exposed to varying doses of x‐rays (0, 4, 8, 10, 14, and 18 Gy) for different durations (0, 6, 24, and 48 h). Cell viability decreased progressively with increasing radiation dose and exposure time (*p* < 0.01, Figure 1A). Based on these results, 10 Gy irradiation for 48 h was selected as the IR condition for subsequent experiments. Compared with the control group, IR‐treated cells showed significantly reduced viability (*p* < 0.01, Figure 1B), increased LDH release (*p* < 0.01, Figure 1C), and a significant increase in γ‐H2AX foci formation (*p* < 0.01, Figure 1D). Moreover, protein levels of NLRP3, Cleaved Caspase‐1, GSDMD‐N, IL‐1β, and IL‐18 were elevated (*p* < 0.01, Figure 1E,F), indicating that x‐ray exposure triggered pyroptosis in HUVECs. IRF1 expression was also significantly upregulated in the IR group (*p* < 0.01, Figure 1E,G), suggesting its involvement in radiation‐induced injury.

To further evaluate the role of IRF1, three siRNAs targeting IRF1 were transfected into HUVECs, all of which effectively reduced IRF1 expression (*p* < 0.01, Figure 1H). Among them, si‐IRF1‐1 and si‐IRF1‐3 demonstrated the highest silencing efficiency and were selected for subsequent experiments. Following irradiation, IRF1 silencing (IR + si‐IRF1 groups) led to significantly decreased IRF1 levels compared with the IR + si‐NC group (Figure 1E,G). This was accompanied by improved cell viability (*p* < 0.01, Figure 1B), reduced LDH release, and fewer γ‐H2AX foci (*p* < 0.01, Figure 1C,D). Furthermore, expression of NLRP3, Cleaved Caspase‐1, GSDMD‐N, IL‐1β, and IL‐18 was significantly decreased (*p* < 0.01, Figure 1E,F). These findings indicate that IRF1 silencing suppresses pyroptosis, thereby mitigating radiation‐induced injury in HUVECs.

### 3.2 IRF1 Binds to the NFE2L1‐DT Promoter and Promotes NFE2L1‐DT Expression

**FIGURE 2:**
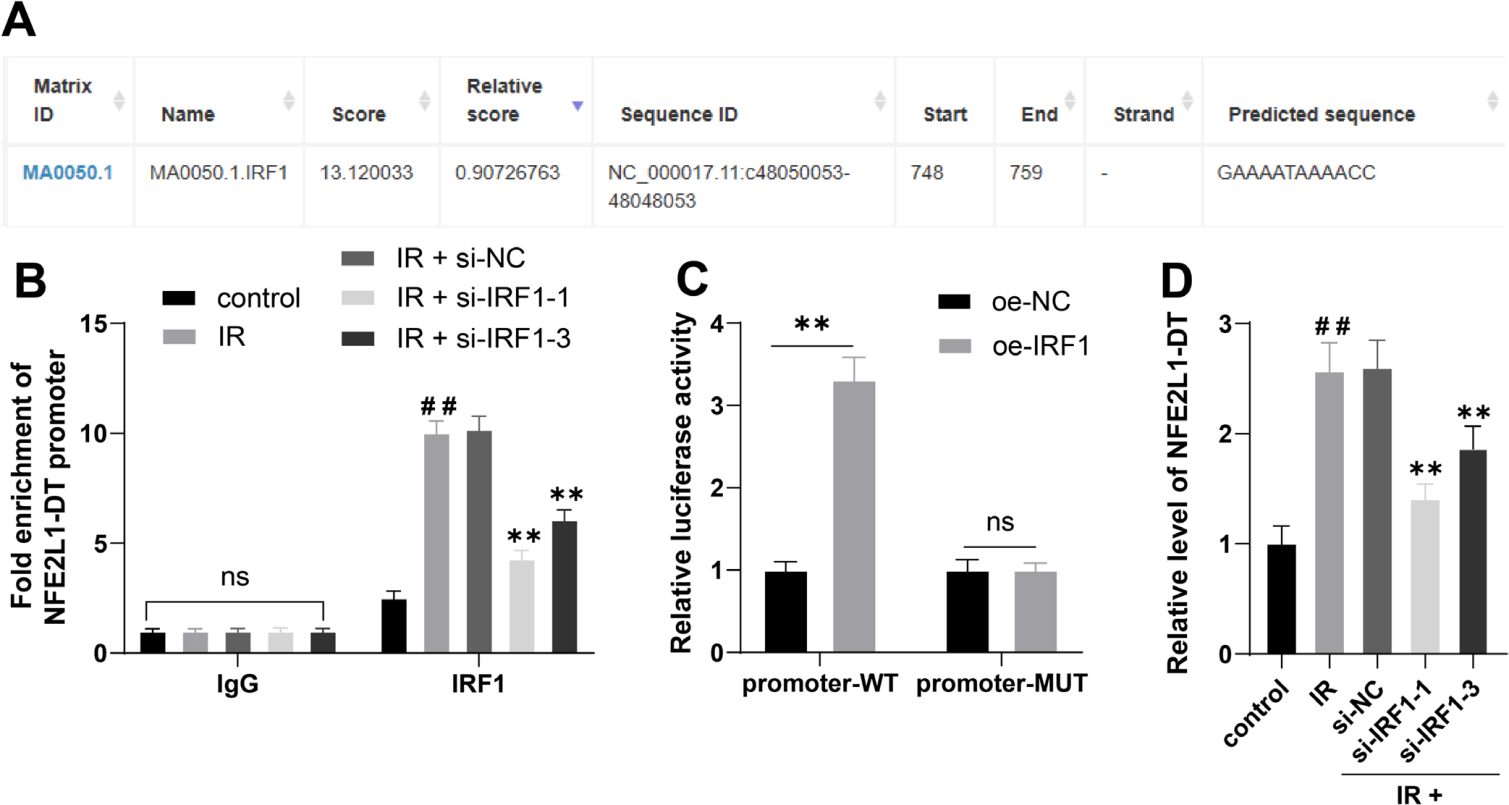
IRF1 binds to the NFE2L1‐DT promoter and upregulates its expression. (A) Predicted IRF1 binding sites within the NFE2L1‐DT promoter region using the JASPAR database. (B) IRF1 enrichment on the NFE2L1‐DT promoter validated by ChIP assay. (C) Interaction between IRF1 and the NFE2L1‐DT promoter confirmed by dual‐luciferase reporter assay. (D) NFE2L1‐DT expression levels determined by RT‐qPCR. Data represent mean ± standard deviation from three independent experiments. For panels B and C, multiple group comparisons were performed using two‐way ANOVA; for panel D, one‐way ANOVA followed by Tukey's post hoc test was applied. Statistical significance: (B–D) ##*p* < 0.01 versus control group; **p < 0.01 versus IR + si‐NC group; (C) ns, *p* > 0.05; **p < 0.01.

Transcription factors can stabilize lncRNA expression by binding to promoter regions [14], and lncRNA NFE2L1‐DT is highly expressed in IR [13], suggesting that IRF1 may regulate cellular radiation injury by affecting NFE2L1‐DT. Therefore, we first used the JASPAR database to predict potential IRF1 binding sites on the NFE2L1‐DT promoter (Figure 2A). ChIP analysis showed that IRF1 binding to the NFE2L1‐DT promoter was significantly enriched in the IR group compared with the control group (*p* < 0.01), whereas IRF1 enrichment was significantly reduced following silencing IRF1 (*p* < 0.01, Figure 2B). Dual‐luciferase reporter assays demonstrated that overexpression of IRF1 significantly increased luciferase activity in the promoter‐WT construct (*p* < 0.01), while no significant change was observed in the promoter‐MUT construct (*p* > 0.05, Figure 2C). Similarly, RT‐qPCR revealed elevated NFE2L1‐DT expression in the IR group compared with controls (*p* < 0.01), which was significantly decreased after IRF1 silencing (*p* < 0.01, Figure 2D). These findings suggest that IRF1 promotes NFE2L1‐DT transcription by directly binding to its promoter.

### 3.3 NFE2L1‐DT Overexpression Attenuates the Inhibitory Effect of IRF1 Knockdown on Radiation‐Induced Injury in HUVECs

**FIGURE 3:**
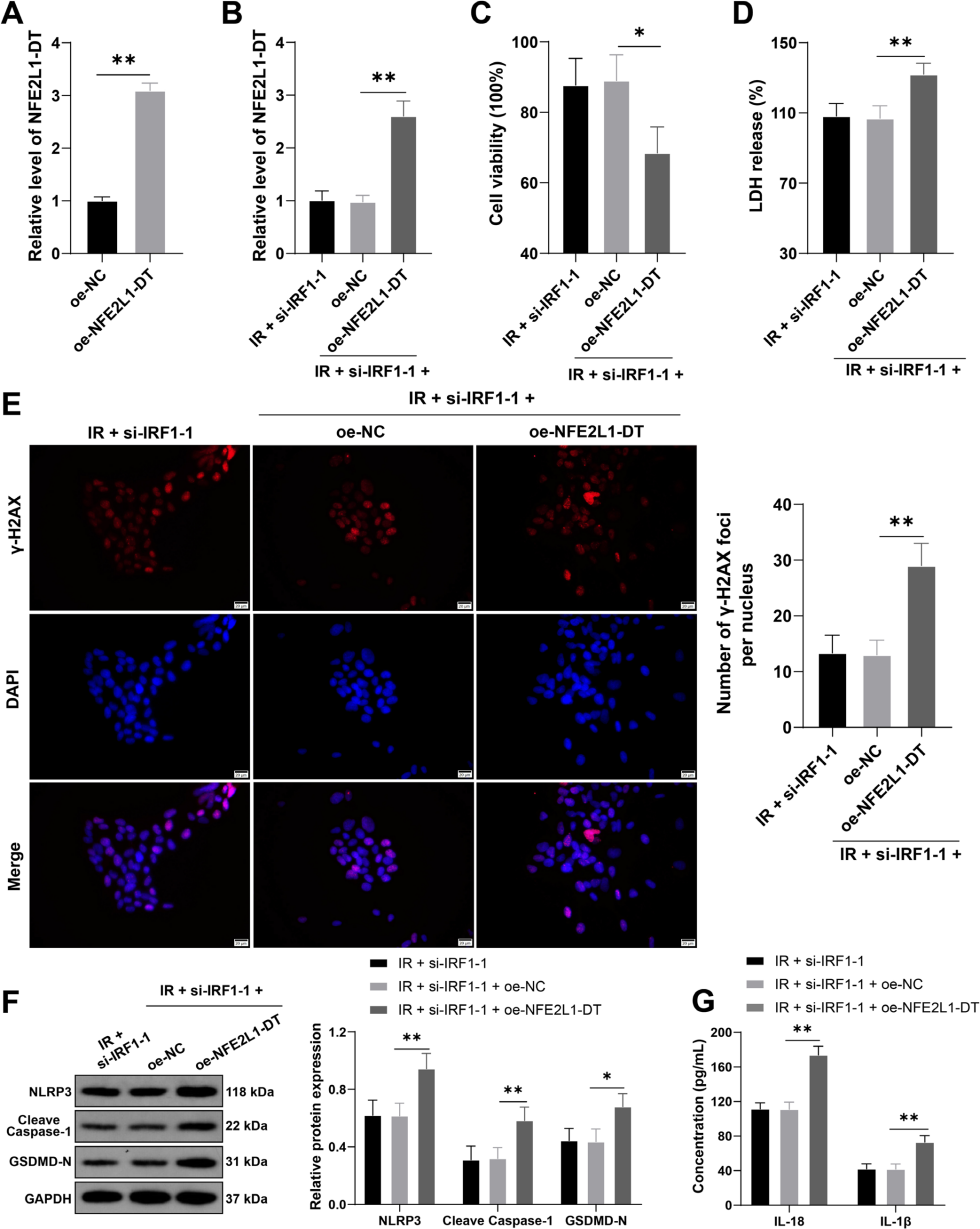
NFE2L1‐DT overexpression reverses the protective effect of silencing IRF1 on radiation‐induced injury in HUVECs. The oe‐NFE2L1‐DT plasmid or empty vector control (oe‐NC) was transfected into HUVECs, followed by treatment with IR + si‐IRF1‐1. (A and B) NFE2L1‐DT expression determined by RT‐qPCR. (C) Cell viability was assessed by the CCK‐8 assay. (D) LDH release was measured using a commercial kit. (E) γ‐H2AX foci quantified by immunofluorescence. (F) Expression of NLRP3, Cleaved Caspase‐1, and GSDMD‐N was evaluated by Western blot. (G) IL‐1β and IL‐18 levels were measured by ELISA. Data are presented as mean ± standard deviation from three independent experiments. Statistical analysis: Panel A analyzed by independent samples *t*‐test; panels B–E analyzed by one‐way ANOVA; panels F and G analyzed by two‐way ANOVA, followed by Tukey's post hoc test. Significance: *p < 0.05; **p < 0.01.

To further confirm the regulatory mechanism, the NFE2L1‐DT overexpression plasmid (oe‐NFE2L1‐DT) was transfected into HUVECs, resulting in a significant increase in NFE2L1‐DT expression (*p* < 0.01, Figure 3A,B). Co‐treatment with IR + si‐IRF1‐1 was then performed. Compared with IRF1 silencing alone, the combination treatment led to reduced cell viability (*p* < 0.05, Figure 3C), elevated LDH release and γ‐H2AX foci (*p* < 0.01, Figure 3D,E), and increased expression of NLRP3, Cleaved Caspase‐1, GSDMD‐N, IL‐1β, and IL‐18 (*p* < 0.01, Figure 3F,G). These findings indicate that the overexpression of NFE2L1‐DT counteracts the protective effect of IRF1 silencing, restoring pyroptosis and aggravating radiation‐induced injury in HUVECs.

### 3.4 NFE2L1‐DT Binds to PELP1 and Promotes the Binding of PELP1 and ALKBH5 mRNA to Upregulate ALKBH5 Expression

**FIGURE 4:**
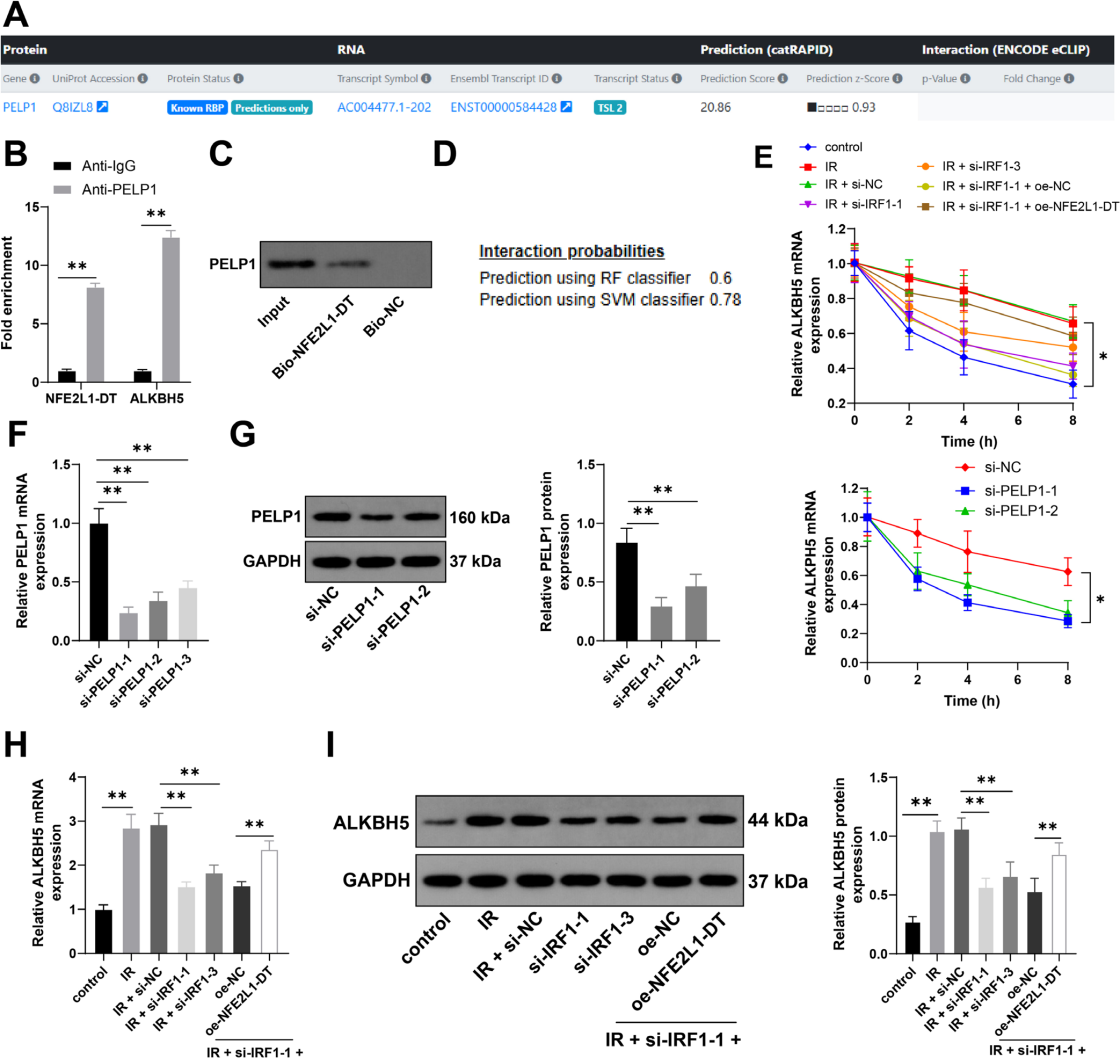
NFE2L1‐DT interacts with PELP1 and enhances the binding of PELP1 and ALKBH5 mRNA to upregulate ALKBH5 expression. (A) Predicted binding interaction between NFE2L1‐DT and PELP1 from the RNAct database. (B) Binding of PELP1 to NFE2L1‐DT or ALKBH5 confirmed by RIP assay. (C) Interaction between PELP1 and NFE2L1‐DT was verified by RNA pull‐down assay, with the input group as a positive control. (D) Predicted binding probability between the PELP1 protein and ALKBH5 from the RPISeq database. (E) ALKBH5 mRNA stability was assessed following actinomycin D treatment. (F and G) PELP1 expression was evaluated by RT‐qPCR and Western blot after transfection of three PELP1‐targeting siRNAs, with si‐NC as a control. (H and I) ALKBH5 expression was assessed by RT‐qPCR and Western blot. Data are presented as mean ± standard deviation from three independent experiments. Statistical analysis: Panels B and E were analyzed by two‐way ANOVA; panels F–I analyzed by one‐way ANOVA, followed by Tukey's post hoc test. Significance: *p < 0.05; **p < 0.01.

The RNAct database predicted a binding interaction between NFE2L1‐DT and the PELP1 protein (Figure 4A). This interaction was experimentally validated by RIP and RNA pull‐down assays, which showed significant enrichment of NFE2L1‐DT with PELP1 (*p* < 0.01, Figure 4B) and successful pull‐down of PELP1 by the biotin‐labeled NFE2L1‐DT probe (Figure 4C). Furthermore, the RPISeq database suggested a high binding probability between PELP1 and ALKBH5 (Figure 4D), which was confirmed by RIP analysis (*p* < 0.01, Figure 4B).

Assessment of ALKBH5 mRNA stability revealed higher stability in the IR group compared with the control group (*p* < 0.05). This stability was reduced upon IRF1 silencing (*p* < 0.05) but restored following NFE2L1‐DT overexpression (*p* < 0.05, Figure 4E). To further investigate the role of PELP1, three siRNAs targeting PELP1 were transfected into HUVECs, effectively silencing its expression (*p* < 0.01, Figure 4F,G). The two siRNAs with the greatest silencing efficiency (PELP1‐1 and PELP1‐2) were used for subsequent experiments, and PELP1 silencing significantly decreased ALKBH5 mRNA stability (*p* < 0.05, Figure 4E).

Similarly, ALKBH5 mRNA and protein levels were elevated in the IR group compared to the control group (*p* < 0.01), reduced after IRF1 silencing (*p* < 0.01), and increased again following NFE2L1‐DT overexpression (*p* < 0.01, Figure 4H,I). These findings suggest that NFE2L1‐DT facilitates ALKBH5 expression through its interaction with PELP1 to enhance ALKBH5 mRNA stability.

### 3.5 ALKBH5 Overexpression Attenuates the Protective Effects of IRF1 Silencing on Radiation‐Induced Injury in HUVECs

**FIGURE 5:**
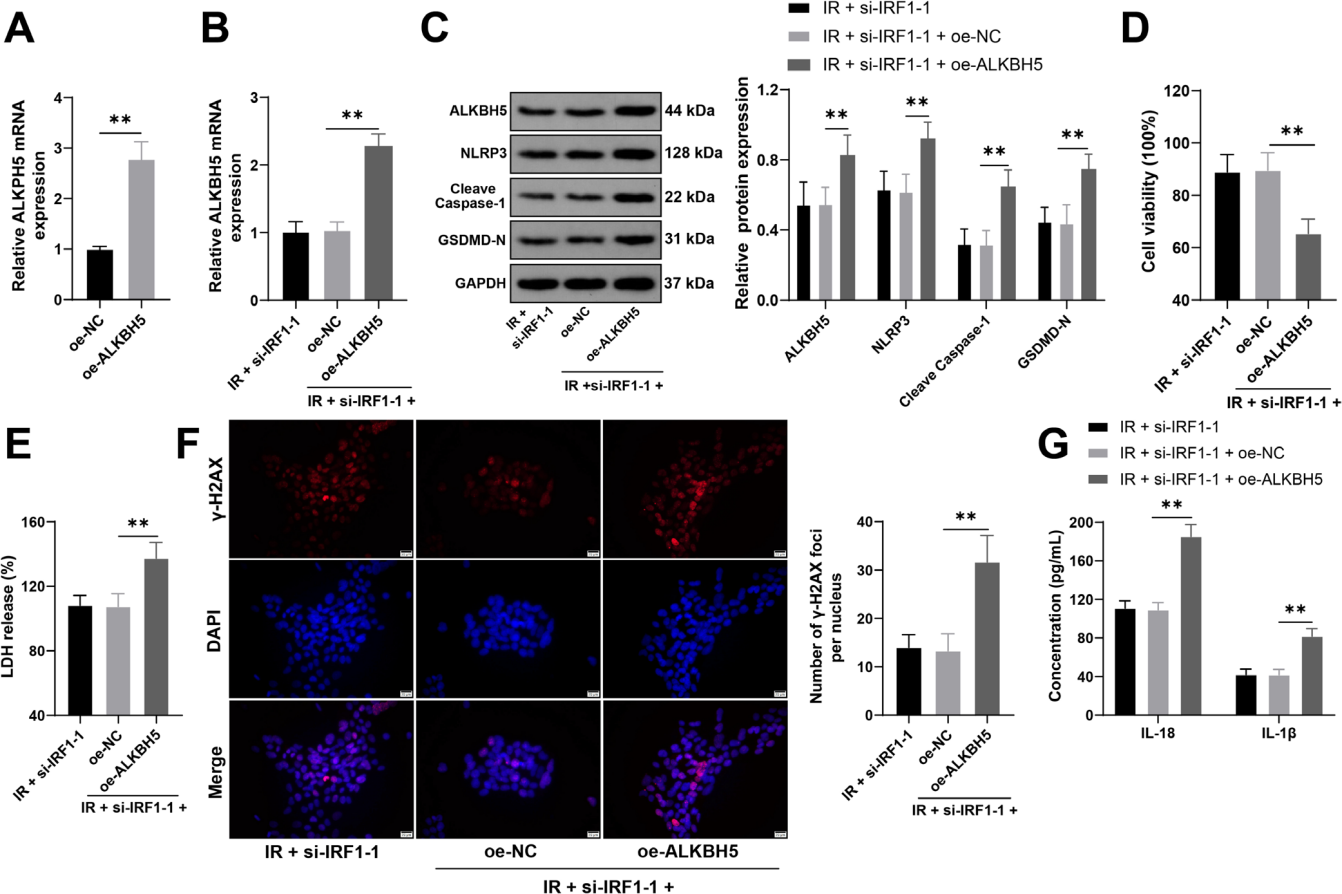
ALKBH5 overexpression counteracts the protective effects of IRF1 silencing on radiation‐induced injury in HUVECs. The ALKBH5 overexpression vector (oe‐ALKBH5) was transfected into HUVECs, with oe‐NC as the control, followed by co‐treatment with IR + si‐IRF1‐1. (A and B) ALKBH5 expression determined by RT‐qPCR. (C) Protein levels of ALKBH5, NLRP3, Cleaved Caspase‐1, and GSDMD‐N were assessed by Western blot. (D) Cell viability was evaluated by the CCK‐8 assay. (E) LDH release was measured using a commercial kit. (F) γ‐H2AX foci quantified by immunofluorescence. (G) IL‐1β and IL‐18 concentrations measured by ELISA. Data are presented as mean ± standard deviation from three independent experiments. Statistical analysis: Panel A analyzed by independent samples *t*‐test; panels B and D–F by one‐way ANOVA; panels C and G by two‐way ANOVA, all followed by Tukey's post hoc test. Significance: **p < 0.01.

To further confirm that IRF1 participates in radiation‐induced injury via ALKBH5, HUVECs were transfected with oe‐ALKBH5 to increase ALKBH5 expression (*p* < 0.01, Figure 5A–C), followed by combined treatment with IR + si‐IRF1‐1. ALKBH5 overexpression significantly decreased cell viability (*p* < 0.01, Figure 5D), increased LDH release and γ‐H2AX foci formation (*p* < 0.01, Figure 5E,F), and elevated the expression of NLRP3, Cleaved Caspase‐1, GSDMD‐N (*p* < 0.01, Figure 5C), as well as IL‐1β and IL‐18 (*p* < 0.01, Figure 5G). These findings demonstrate that ALKBH5 overexpression reverses the protective effects of IRF1 silencing, restoring pyroptosis and exacerbating radiation‐induced injury in HUVECs.

### 3.6 ALKBH5 Reduces Cx43 Stability by Demethylating m^6^A Modifications

**FIGURE 6:**
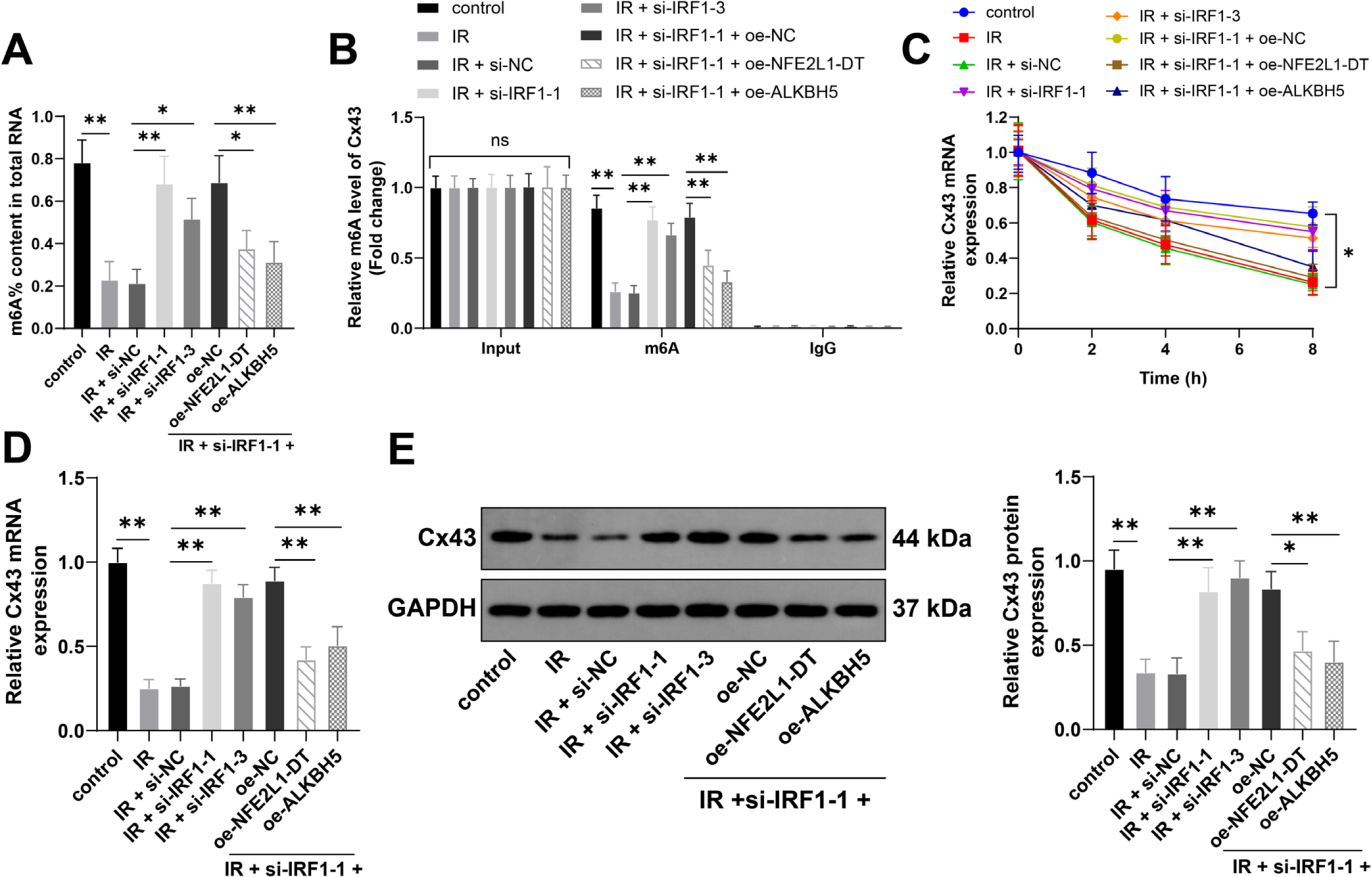
ALKBH5 reduces Cx43 stability by demethylating m^6^A modifications. (A) Total m^6^A content measured in HUVECs. (B) m^6^A enrichment on Cx43 detected by MeRIP‐qPCR. (C) Cx43 mRNA stability was assessed following actinomycin D treatment. (D) Cx43 mRNA expression measured by RT‐qPCR. (E) Cx43 protein expression determined by Western blot. Data are presented as mean ± standard deviation from three independent experiments. Statistical analysis: Panels A, D, and E analyzed by one‐way ANOVA; panels B and C analyzed by two‐way ANOVA, all followed by Tukey's post hoc test. Significance: ns, *p* > 0.05; *p < 0.05; **p < 0.01.

Total m^6^A content in cells was significantly lower in the IR group compared to the control group (*p* < 0.01). Silencing IRF1 significantly increased m^6^A levels (*p* < 0.05), while overexpression of NFE2L1‐DT or ALKBH5 reversed this increase (*p* < 0.05, Figure 6A). MeRIP‐qPCR analysis showed reduced m^6^A enrichment on Cx43 mRNA in the IR group (*p* < 0.01), which was restored by IRF1 silencing (*p* < 0.01) and subsequently diminished following NFE2L1‐DT or ALKBH5 overexpression (*p* < 0.01, Figure 6B). Cx43 mRNA stability was lower in the IR group (*p* < 0.05), increased after IRF1 silencing (*p* < 0.05), and decreased again with NFE2L1‐DT or ALKBH5 overexpression (*p* < 0.05, Figure 6C). Similarly, RT‐qPCR and Western blot results confirmed that Cx43 expression was downregulated in the IR group (*p* < 0.01), upregulated after IRF1 silencing (*p* < 0.01), and reduced again upon NFE2L1‐DT or ALKBH5 overexpression (*p* < 0.05, Figure 6D,E). These results suggest that ALKBH5 decreases Cx43 stability by removing m^6^A modifications.

### 3.7 Cx43 Silencing Attenuates the Inhibitory Effect of IRF1 Silencing on Radiation‐Induced Injury in HUVECs

**FIGURE 7:**
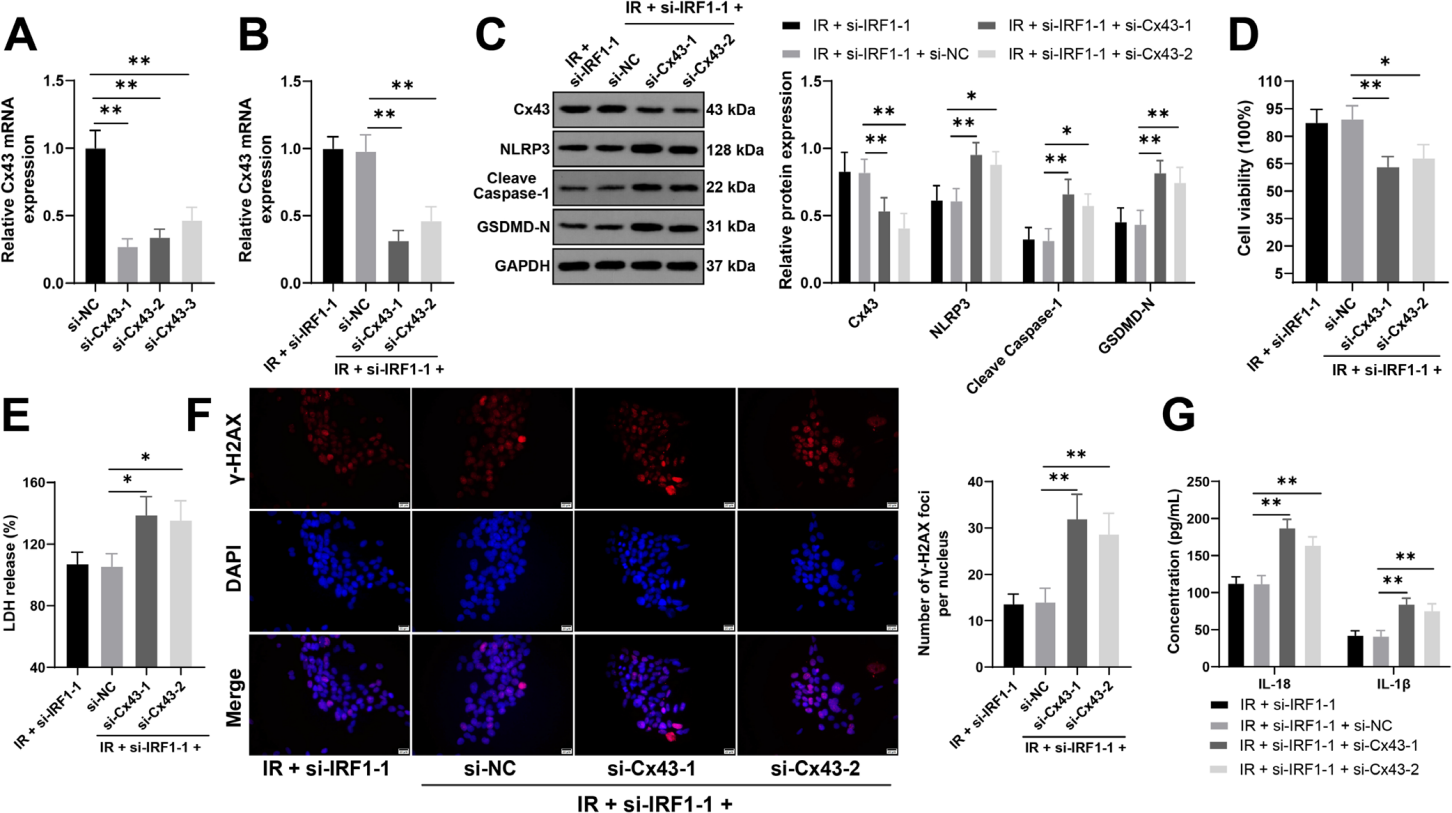
Cx43 silencing mitigates the reversal of IRF1 silencing on radiation‐induced injury in HUVECs. Three siRNAs targeting Cx43 were transfected into HUVECs, with si‐NC as the control, followed by co‐treatment with IR + si‐IRF1‐1. (A and B) Cx43 mRNA expression measured by RT‐qPCR. (C) Protein expression of Cx43, NLRP3, Cleaved Caspase‐1, and GSDMD‐N was assessed by Western blot. (D) Cell viability measured by CCK‐8 assay. (E) LDH release was determined using a commercial kit. (F) γ‐H2AX foci detected by immunofluorescence. (G) IL‐1β and IL‐18 levels quantified by ELISA. Data are presented as mean ± standard deviation from three independent experiments. Statistical analysis: Panels A, B, and D–F analyzed by one‐way ANOVA; panels C and G analyzed by two‐way ANOVA, followed by Tukey's post hoc test. Significance: *p < 0.05; **p < 0.01.

To further validate this mechanism, three siRNAs targeting Cx43 were introduced into HUVECs to silence its expression (*p* < 0.01, Figure 7A). Among them, si‐Cx43‐1 and si‐Cx43‐2, which showed higher silencing efficiency, were selected for subsequent experiments in combination with IR + si‐IRF1‐1 treatment. Compared with the IR + si‐IRF1‐1 + si‐NC group, the combination group showed significantly reduced Cx43 expression (*p* < 0.01, Figure 7B,C), along with decreased cell viability (*p* < 0.05, Figure 7D), increased LDH release and γ‐H2AX foci formation (*p* < 0.05, Figure 7E,F), and elevated levels of NLRP3, Cleaved Caspase‐1, GSDMD‐N (*p* < 0.05, Figure 7C), IL‐1β, and IL‐18 (*p* < 0.01, Figure 7G). In summary, Cx43 silencing counteracts the protective effect of IRF1 silencing, improving pyroptosis and radiation‐induced injury in HUVECs.

## 4 Discussion

**FIGURE 8:**
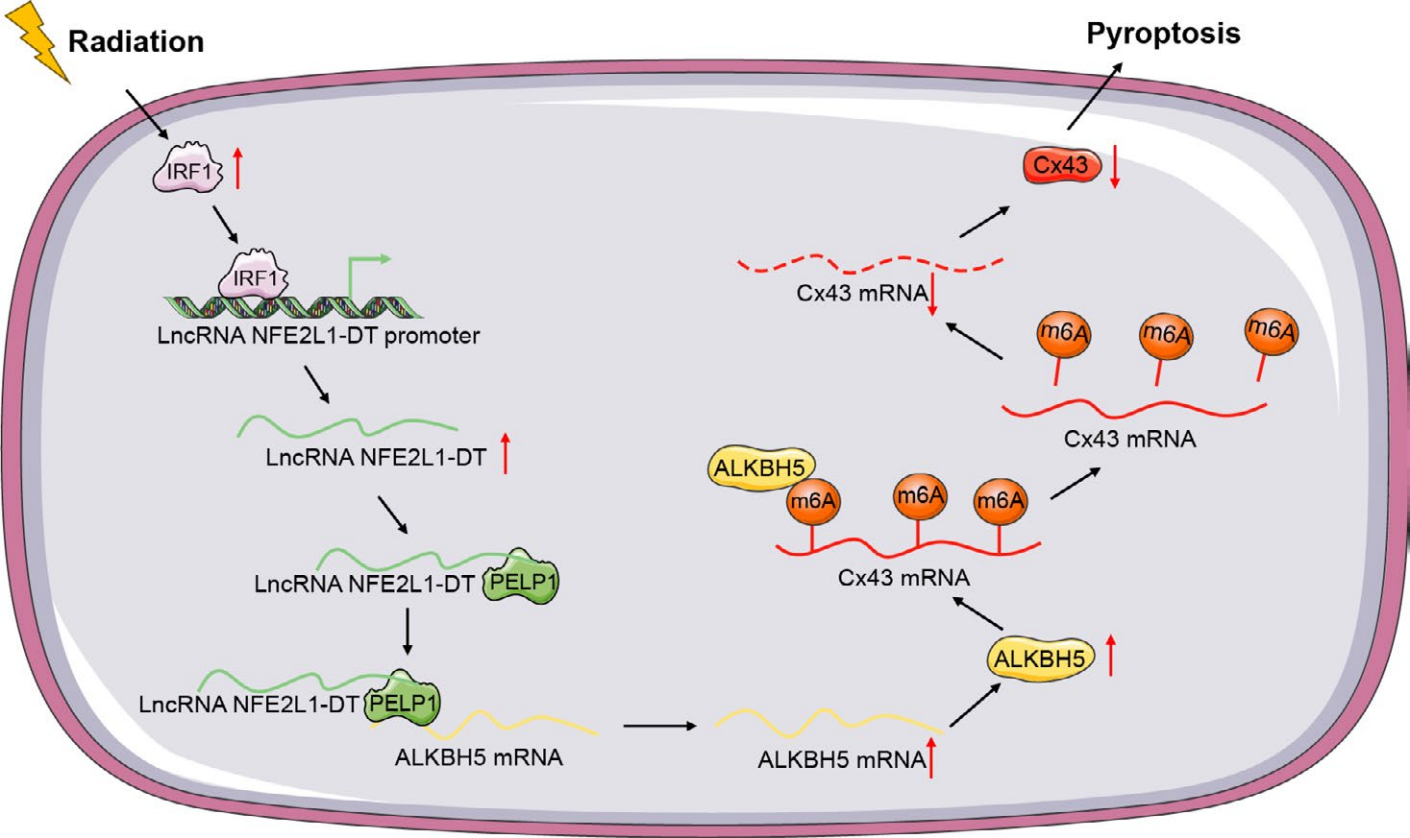
Proposed mechanism by which radiation induces pyroptosis in HUVECs via the IRF1–NFE2L1‐DT–ALKBH5–Cx43 axis. Radiation exposure increases IRF1 expression, which transcriptionally activates NFE2L1‐DT. NFE2L1‐DT interacts with PELP1 to enhance the binding of PELP1 and ALKBH5 mRNA and promote ALKBH5 mRNA stability, thus upregulating ALKBH5 expression. ALKBH5 reduces m^6^A methylation of Cx43 mRNA, leading to decreased Cx43 expression and facilitating pyroptosis in HUVECs.

As a cornerstone treatment for cancer, radiotherapy can cause both microvascular and macrovascular damage by injuring endothelial cells, which may ultimately lead to cardiovascular complications [24]. In this study, we established an endothelial cell radiation injury model by exposing HUVECs to x‐ray irradiation, which resulted in a pronounced increase in IRF1 expression. IRF1 transcriptionally upregulated NFE2L1‐DT, which in turn interacted with PELP1 to increase the transcription and expression of ALKBH5. ALKBH5 reduced m^6^A modification of Cx43 mRNA, leading to decreased Cx43 expression, promoting pyroptosis and exacerbating radiation‐induced endothelial injury (Figure 8).

A significant upregulation was first observed for IRF1 expression, accompanied by a significant increase in pyroptosis levels in radiation‐treated HUVECs. IR‐induced mitochondrial DNA leakage has been shown to drive IRF1 nuclear translocation, activating inflammatory responses and promoting cell death [8]. Silencing IRF1 led to a pronounced reduction in NLRP3, Cleaved Caspase‐1, GSDMD‐N, IL‐1β, and IL‐18 expression, resulting in attenuation of pyroptosis. As a transcription factor, IRF1 directly binds to the GSDMD promoter and enhances its transcription, facilitating NLRP3‐mediated endothelial pyroptosis and contributing to the progression of atherosclerosis [25]. Similarly, in high glucose‐induced pyroptosis of retinal endothelial cells, IRF1 knockdown has been reported to suppress the expression of Caspase‐1, GSDMD, and IL‐1β [26]. These findings, together with our data, suggest a broad pro‐pyroptotic role of IRF1 in endothelial cells. Radiotherapy can upregulate IRF1 expression and induce ferroptosis in head and neck squamous cell carcinoma, thus impeding tumor progression [27]. In mouse colon cancer cells, IRF1 knockdown has been shown to inhibit GSDMD activation and limit pyroptosis [28]. Given IRF1's pro‐pyroptotic effects in both tumor and endothelial cells, therapeutic approaches must be precisely tailored. The development of endothelium‐specific IRF1 inhibitors could minimize systemic toxicity and improve the therapeutic index [29]. In summary, IRF1 silencing emerges as a promising strategy to mitigate radiation‐induced endothelial injury, with endothelial‐targeted IRF1 inhibitors holding significant clinical potential in cancer radiotherapy.

IRF1 was found to bind to the NFE2L1‐DT promoter, stabilizing its expression. Overexpression of NFE2L1‐DT in HUVECs led to increased levels of pyroptosis markers and a higher number of γ‐H2AX foci. LncRNAs have emerged as important therapeutic targets in regulating pyroptosis in cardiovascular diseases, which constitute a major adverse effect of radiotherapy [30]. For instance, lncRNA Gaplinc interacts with SP1 to upregulate NLRP3 expression, promoting endothelial cell pyroptosis and driving the expansion of atherosclerotic plaques in high‐fat diet‐fed mice [31]. Similarly, lncRNA H19 overexpression elevates IL‐1β, IL‐18, IL‐6, and LDH levels in aortic endothelial cells, contributing to hyperlipidemia and exacerbating atherosclerosis in mice [32]. The interplay between lncRNAs and RNA‐binding proteins represents a key regulatory mechanism in cancer therapy [33]. In our study, RIP and RNA pull‐down assays demonstrated that NFE2L1‐DT increases ALKBH5 expression by interacting with PELP1, which in turn elevates the expression of NLRP3, Cleaved Caspase‐1, GSDMD‐N, IL‐1β, and IL‐18, aggravating radiation‐induced endothelial injury.

In radiation‐exposed HUVECs, we observed a significant reduction in global m^6^A levels, with ALKBH5 showing a negative regulatory relationship with m^6^A content. Recent evidence indicates that in ropivacaine‐induced pyroptosis of HUVECs, dexmedetomidine decreases ALKBH5 expression, leading to increased m^6^A modification of FUNDC1 mRNA and suppression of inflammation and pyroptosis [34]. Moreover, ALKBH5 has been reported to mediate m^6^A modification of NLRP3 mRNA in an IGF2BP2‐dependent manner, establishing a positive feedback loop that activates cardiomyocyte pyroptosis [35]. In our study, we confirmed that ALKBH5 demethylates Cx43 mRNA, reducing its stability and expression. Cx43 silencing, in turn, elevated pyroptosis levels and decreased cell viability. Consistent with our findings, overexpression of Cx43 in x‐ray‐treated HUVECs has been shown to lower intracellular calcium concentration and attenuate pyroptosis [36], and further, Cx43 overexpression can inhibit caspase‐1 activation and reduce cleaved Panx1, preventing pyroptosis in x‐ray irradiated HUVECs [20]. These results indicate that ALKBH5 downregulates Cx43 expression in an m^6^A‐dependent manner, reinstating pyroptosis in radiation‐treated HUVECs.

The mechanisms underlying radiation‐induced vascular endothelial injury are multifaceted, and we acknowledge several limitations in our current study. First, although our findings demonstrate that IRF1 regulates endothelial radiation injury via pyroptosis, other pathways may also be involved. As a transcription factor, IRF1 modulates a broad array of immune and stress response genes, yet in this study, we focused exclusively on NFE2L1‐DT as its downstream target. Second, our conclusions have not yet been corroborated by in vivo or clinical studies. The absence of animal model validation and clinical data restricts the translational potential and practical applicability of our results. Third, although we observed Cx43 downregulation in radiation‐injured HUVECs, previous studies have reported increased Cx43 expression in radiation‐injured cardiomyocytes and astrocytes, suggesting that the regulation of Cx43 may be cell type specific and context‐dependent [37, 38]. The variable expression of Cx43 across different radiation‐exposed cell types may reflect its multifunctional roles, including modulation of osmotic pressure, regulation of mitochondrial activity, and alterations in membrane architecture [38, 39]. The mechanisms underlying the differential expression of Cx43 in radiation injury across various cell types and disease contexts remain to be elucidated. Fourth, our identification of pyroptosis based on NLRP3, Cleaved Caspase‐1, GSDMD‐N, IL‐1β, and IL‐18 may overlap with other forms of cell death, such as PANoptosis, necroptosis, or inflammasome‐independent inflammatory responses. Lastly, it remains to be determined whether independent modulation of NFE2L1‐DT or ALKBH5 can trigger HUVEC pyroptosis in the absence of IRF1. Future studies will aim to explore other downstream pathways of IRF1 and incorporate clinical investigations to provide stronger theoretical support for improving patient outcomes following radiotherapy.

In conclusion, radiation‐induced IRF1 upregulation leads to pyroptosis and drives vascular endothelial injury through the NFE2L1‐DT/ALKBH5/Cx43 axis. These findings highlight potential therapeutic targets for the prevention and management of radiotherapy‐associated complications.

## Conflicts of Interest

The authors declare no conflicts of interest.

## Data Availability

The data that support the findings of this study are available from the corresponding author upon reasonable request.
